# A conserved SH3-like fold in diverse putative proteins tetramerizes into an oxidoreductase providing an antimicrobial resistance phenotype

**DOI:** 10.1098/rstb.2022.0040

**Published:** 2023-02-27

**Authors:** Claudèle Lemay-St-Denis, Lorea Alejaldre, Zakaria Jemouai, Kiana Lafontaine, Maxime St-Aubin, Katia Hitache, Donya Valikhani, Nuwani W. Weerasinghe, Myriam Létourneau, Christopher J. Thibodeaux, Nicolas Doucet, Christian Baron, Janine N. Copp, Joelle N. Pelletier

**Affiliations:** ^1^ PROTEO, The Québec Network for Research on Protein, Function, Engineering and Applications, Québec, Qué‌bec G1V 0A6, Canada; ^2^ CGCC, Center in Green Chemistry and Catalysis, Montréal, Québec‌ H2V 0B3, Canada; ^3^ Department of Biochemistry and Molecular Medicine, Université de Montréal, Montréal, Québec‌ H3C 3J7, Canada; ^4^ Chemistry Department, Université de Montréal, Montréal, Québec‌ H3C 3J7, Canada; ^5^ Department of Microbiology, Infectiology and Immunology, Université de Montréal, Montréal, Québec‌ H3C 3J7, Canada; ^6^ Department of Chemistry and Centre de Recherche en Biologie Structurale, McGill University, Montréal, Québec H3A 0B8, Canada; ^7^ Centre Armand-Frappier Santé Biotechnologie, Institut National de la Recherche Scientifique (INRS), Université du Québec, Laval, Québec‌ H7V 1B7, Canada; ^8^ Michael Smith Laboratories, University of British Columbia, Vancouver, British Columbia V6T 1Z4, Canada

**Keywords:** evolutionary biochemistry, enzyme evolution, R67 DHFR, antibiotic resistance, SH3-like fold

## Abstract

We present a potential mechanism for emergence of catalytic activity that is essential for survival, from a non-catalytic protein fold. The type B dihydrofolate reductase (DfrB) family of enzymes were first identified in pathogenic bacteria because their dihydrofolate reductase activity is sufficient to provide trimethoprim (TMP) resistance. DfrB enzymes are described as poorly evolved as a result of their unusual structural and kinetic features. No characterized protein shares sequence homology with DfrB enzymes; how they evolved to emerge in the modern resistome is unknown. In this work, we identify DfrB homologues from a database of putative and uncharacterized proteins. These proteins include an SH3-like fold homologous to the DfrB enzymes, embedded in a variety of additional structural domains. By means of functional, structural and biophysical characterization, we demonstrate that these distant homologues and their extracted SH3-like fold can display dihydrofolate reductase activity and confer TMP resistance. We provide evidence of tetrameric assembly and catalytic mechanism analogous to that of DfrB enzymes. These results contribute, to our knowledge, the first insights into a potential evolutionary path taken by this SH3-like fold to emerge in the modern resistome following introduction of TMP.

This article is part of the theme issue ‘Reactivity and mechanism in chemical and synthetic biology’.

## 1. Introduction

How does enzyme function arise to ensure host survival when faced with metabolic stress? There are many examples of ancient and modern evolutionary paths that have given rise to resistance mechanisms in response to xenobiotic compound exposure [1–5]. The best-described mechanism for evolution of new enzyme function is the duplication of promiscuous enzymes followed by their divergence to improve an activity required for survival [6–10]. Catalytic specialization from promiscuous enzymes results from increased affinity, active-site rearrangement, allosteric changes and altered protein dynamics [11–15]. Examples span from the evolution of β-lactamases from DD-peptidases 3 billion years ago [1,16,17] to the recent evolution of the AtzA atrazine dechlorinase from TriA melamine deaminase following introduction of the synthetic pesticide atrazine into the environment [3].

By contrast, the emergence of catalytic activity that is essential for survival from a non-catalytic protein fold is rare, with few documented examples [18,19]. Nonetheless, the evolution of efficient and stereospecific catalysts from non-catalytic binding proteins has been demonstrated [20–22]. The evolution of a chalcone isomerase from a non-catalytic ancestral protein has been recapitulated, where the successive inclusion of ‘founder’ mutations at the binding site conferred increasing, initial activity followed by progressive modification of distal residues for fine-tuning purposes [23]. Similarly, successive introduction of catalytic residues in the binding site of an ancestral solute-binding protein yielded cyclohexadienyl dehydratase activity that improved upon reshaping by remote substitutions [24]. In another example, evolution of the organomercurial lyase activity of MerB occurred through gene duplication and diversification of the non-enzymatic metallochaperone NosL, which has a related metal-binding function [25,26].

Here, we present evidence supporting a further example of evolution of a binding domain into a catalyst. The NADPH-dependent reductase activity of the type B dihydrofolate reductases (DfrB) enables resistance against the antimicrobial trimethoprim (TMP) [27]. As opposed to many antimicrobials such as the β-lactams and aminoglycosides, TMP is an entirely synthetic molecule that was clinically introduced in the 1960s and has since been used worldwide in clinical, veterinary and livestock industry settings, including application in preventive measures [28–30]. As a result, the wide environmental dissemination of TMP has rapidly given rise to the appearance of resistance mechanisms [28].

DfrB enzymes are formed by homotetramerization of an SH3-like fold, consisting of a 60-residue *ß*-barrel. SH3-like folds are highly versatile and typically bind molecules ranging from peptides and metals to DNA and RNA [31,32]. SH3-like folds have been reported within more than a dozen prokaryotic proteins with diverse binding functions, including protein–protein mediation in the antirepressor CarS [33] and DNA binding in HIV integrase [34]. SH3-like folds have rarely been described to possess catalytic function; to the best of our knowledge, the only examples reported are the type 1 signal peptidases and DfrB enzymes [32].

Evidence supporting the hypothesis that DfrB enzymes may have evolved from a non-catalytic fold is found in their unusual—even qualified as primitive [35]—catalytic mechanism. Whereas the productive binding constants (*K*_M_) of DfrB enzymes are physiologically relevant, the hydride transfer rate of 1.3 s^−1^ qualifies it as slow among enzymes involved in nucleotide metabolism [36,37]. As a result, their catalytic efficiencies are two orders of magnitude lower than the ubiquitous microbial FolA dihydrofolate reductases that are the target of TMP [35,38].

While 10 members of the DfrB family have been reported [27], the evolutionary path that has brought DfrB enzymes to the modern resistome is unclear. Aside from sharing the same catalytic function, the homotetrameric, 60-residue β-barrel DfrB enzymes have no structural or evolutionary properties in common with other known dihydrofolate reductases (Dfr), since the ubiquitous FolA and their homologues are monomers of 150 to 190 residues, belonging to the *α*/*β* class of proteins (electronic supplementary material, figures S1 and S2) [39,40].

Here, we identify and characterize DfrB homologues to provide early insight into the evolution of an SH3-like fold that provides a powerful antimicrobial resistance mechanism. We have identified 30 proteins exhibiting significant sequence homology to the well-characterized DfrB1, by means of searches in a database of predicted and uncharacterized proteins. Characterization of five putative homologues sharing 10–80% global sequence similarity with DfrB1 revealed that four of these homologues catalysed dihydrofolate reduction and conferred strong TMP resistance. Biophysical and kinetic characterization of the most active distant homologue DfrB-H5 suggest the conservation of multimerization and catalytic mechanism with the DfrB family. This work unveils a potential mechanism by which an SH3-like fold procures catalytic activity that has become essential for survival in the recent context of exposure to TMP.

## 2. Results

**Figure 1.**
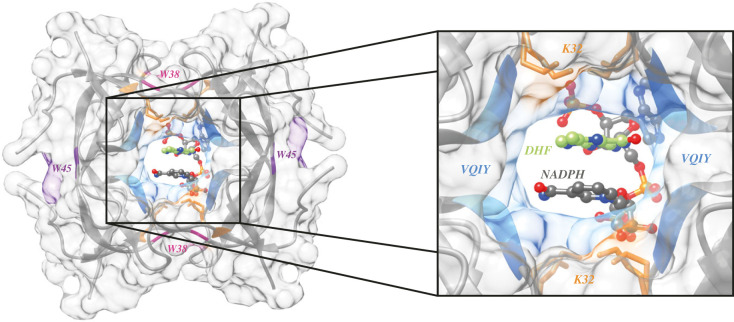
Overview of DfrB1 structure and key residues. The DfrB1 homotetramer (PDB 2RK1) complexed with DHF substrate (carbons in green, only the pterin group is resolved) and NADP^+^ (carbons in grey). The VQIY active-site motif is coloured (from dark to light blue) for each protomer. K32 residues that bind the negative charges of DHF and NADPH are shown as orange sticks (two conformations are shown). Residues W45 at the monomer–monomer interfaces and W38 at the dimer–dimer interfaces, are coloured in purple and pink, respectively. (Online version in colour.)

DfrB1, the best-characterized member of the 10-member DfrB family (electronic supplementary material, figure S3), is active as an obligate homotetramer, where all four protomers participate in forming the enzyme's central, highly symmetrical active site (figure 1). This voluminous, hourglass-shaped active-site tunnel [35] accommodates the substrate dihydrofolate (DHF) and the reducing cofactor NADPH. The active-site residues are known as the conserved VQIY motif [38], spanning from V66 to Y69 on the B4 strand. These residues, which are not directly involved in the catalytic event, form a hydrogen-bonded network both at the floor and ceiling of the active site, forming binding sites that properly orient the reactive groups on DHF and NAPDH for the hydride transfer event by a proximity-based catalytic mechanism (electronic supplementary material, figure S4) [41]. The putative lack of transition state stabilization by active-site residues is again suggestive of a primitive, or poorly evolved, catalytic mechanism.

The symmetrical and tetrameric organization of the active site make the binding site promiscuous; DHF and NADPH occupy an identical space in the central tunnel. The tunnel has four identical surfaces (figure 1) which together form a single functional active site [41]. Thus, a single substitution of an active-site residue results in the simultaneous modification of all four ‘faces’ of the active-site cavity, such that point mutations at the active site of DfrB1 are largely deleterious [42–44]. This poses a clear disadvantage with respect to natural evolution of a highly adapted catalyst. Nonetheless, because there is no direct catalytic involvement of any residue, fully functional variants of DfrB1 have been engineered where three or all four of the VQIY-motif residues were substituted [44]. That study confirmed that none of the VQIY residues is strictly essential, and that catalysis requires a protein environment conducive of the direct hydride transfer from NADPH to DHF.

### (a) Identification of distant homologues of the DfrB enzymes

The protomer unit of DfrB1 is composed of one highly conserved SH3-like fold within the DfrB family, preceded by a poorly conserved, unstructured N-terminus (electronic supplementary material, figure S3). This SH3-like fold has no evolutionary homology to any characterized protein. No distant homologues in the UniProtKB/Swiss-Prot database of functionally annotated sequences are identified for DfrB1 according to PSI-BLAST, using an E-value threshold of 10^−3^; by contrast, applying the same method to the *Escherichia coli* FolA identifies 90 characterized sequences sharing less than 50% local identity.

**Figure 2.**
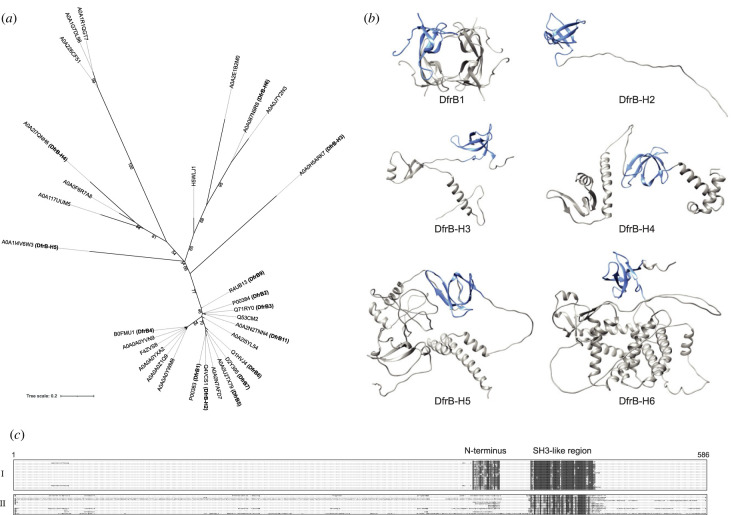
Distant homologues of DfrB. (*a*) Phylogenetic tree (in black) of the 30 non-redundant sequences homologous to DfrB1 in the UniProtKB database was constructed using an alignment of the SH3-like region (positions 24–78 in DfrB1). UniProt IDs are identified for every sequence and connected to their phylogenetic branch with a light grey linker. Bootstrap values are indicated for the branches that separate the main clusters. Nine known DfrB and the five DfrB-H selected for characterization are annotated in bold. (*b*) DfrB1 (PDB 1VIE) homotetramer and DfrB-H structures predicted using ColabFold [45]. The SH3-like folds are coloured in blue in all proteins. (*c*) Alignment of the 30 sequences. Residues are coloured in grey when identical to the consensus sequence. Cluster I is composed of sequences closely related to DfrB1, while cluster II is composed of distantly related sequences. The alignment was generated by MAFFT [46] and represented by UGENE [47]. The full alignment is presented in the electronic supplementary material, figure S5. (Online version in colour.)

Recognizing that standard search tools are inefficient in yielding evolutionary insight into the emergence of DfrB enzymes, we searched the complete UniProtKB for DfrB homologues, including uncharacterized proteins. By those means, we identified a total of 68 sequences. They describe 30 non-redundant proteins having sequence similarity with DfrB1 among which 21 are described as putative, as they have only been identified by bioinformatic predictions. Their length ranges from 67 to 463 amino acids. A set of 18 close homologues to the DfrB1 (greater than 80% global sequence similarity) includes nine of the 10 known DfrB family members; the remaining 12 homologues are more distantly related (figure 2*a,c*). We selected five homologues of DfrB1 for characterization, which we named DfrB-H2 to DfrB-H6: one close homologue to DfrB1 (DfrB-H2, global similarity of 80%) and four more distantly related homologues with lower global sequence similarity (between 10 and 31%) owing largely to the presence of a diverse array of additional domains (electronic supplementary material, figures S6 and S7). These DfrB-H proteins are 97 to 463 residues in length (electronic supplementary material, table S1).

DfrB-H2, the closest homologue (figure 2*a*), has been reported in a number of bacterial contexts. Here, we identified a sequence from *Klebsiella pneumoniae* in the genomic context of other genes involved in antibiotic resistance, including the metallo-β-lactamase *blaVIM-1*, the aminoglycoside acetyltransferase *aacA4* and the chloramphenicol acetyltransferase *catB2*. While the 58-residue SH3-like core is essentially identical to DfrB1 (a single substitution), the 39-residue N-terminus of DfrB-H2 is twice the length and includes the DfrB1 N-terminus. Although the other nine reported DfrB are always identified as 78 residues in length, this gene has two start codons: one for the translation of the 97-residue DfrB-H2, and one for a 78-residue enzyme that is identical to DfrB1 except for one residue at the junction of the N-terminus and the SH3-like fold. We have no information on the relative expression levels of each gene product *in vivo*. In fact, the well-studied, ‘canonical’ DfrB1 may not be produced in nature in its 78-residue form but may always be accompanied by the longer N-terminus that characterizes DfrB-H2. Characterization of DfrB-H2 will inform on the impact of varying the N-terminus length on DfrB function.

The four more distant homologues, DfrB-H3 to DfrB-H6, are all mainly clustered among genes encoding hypothetical proteins having no predicted function. Among the few genes having a putative function, both DfrB-H3, identified in a *Pseudomonas* phage, and DfrB-H5, identified in *Methylobacterium pseudosasicola*, are found in proximity to a DNA methyltransferase. DfrB-H4, identified in a *Vibrio* phage, is found at a distance of 2 kb of a DNA methylase gene. Finally, DfrB-H6, identified in a *Spingobium* species, is 1.1 kb from a gene for a tyrosine-like recombinase, 1.5 kb from a transcriptional regulator of the LysR family and 2.8 kb from an endonuclease. While the predicted functions of those surrounding genes are related to DNA modification, the endogenous function of each DfrB-H remains unknown.

Structure prediction for these distant homologues suggests the presence of an SH3-like fold in each of the five homologues (figure 2*b*). The additional domains in each of the distant homologues share no structural or sequence similarity (electronic supplementary material, figure S8). The SH3-like folds share structural similarity as well as high sequence similarity (67–100%) and identity (55–98%; electronic supplementary material, figure S7), suggesting common ancestry. Notably, the active-site VQIY motif along with the K32 and W38 residues, required for substrate binding and for tetramerization, respectively (figure 1), is conserved in all DfrB-H (electronic supplementary material, figure S8). However, the W45 monomer–monomer interface residue of DfrB1 (figure 1), that can be substituted with no significant modification of structure or catalytic function [48], is not conserved.

### (b) Investigation of the DfrB-like phenotype in the DfrB-H

**Figure 3.**
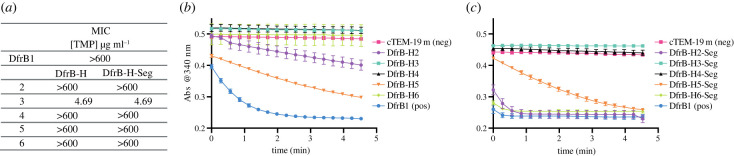
DfrB-H proteins display a DfrB1-like phenotype. (*a*) MIC assays were performed on *E. coli* on Luria-Bertani (LB)-agar IPTG induction media in triplicate. TMP concentration ranged between 4.7 and 600 µg ml^−1^, the highest soluble concentration. (*b*,*c*) Dihydrofolate reductase activity in crude *E. coli* lysate following overexpression of the (*b*) DfrB-H and the (*c*) DfrB-H-Seg, segments encoding the predicted SH3-like fold of the DfrB-H. The fastest reaction rates are seen where substrate depletion has occurred even at the initial time point. An absorbance that remains high and constant is indicative of non-detectable dihydrofolate reductase activity. Assays were performed in triplicate and error bars represent s.d. (Online version in colour.)

For the DfrB-H to display dihydrofolate reductase activity according to the same mechanism as DfrB1, their SH3-like fold must assemble into the homotetramer that characterizes DfrB1, thereby forming the active-site tunnel. The distant homologues include at least two domains other than the SH3-like fold; as a result, tetrameric assembly might be hindered or precluded. We performed minimal inhibitory concentration (MIC) assays to investigate whether the DfrB-H proteins confer a TMP resistance phenotype consistent with dihydrofolate reductase activity when overexpressed in *E. coli*. We note that DfrB1 and the DfrB-H assayed in this study carried an N-terminal His_6_ tag. Surprisingly, DfrB-H2, DfrB-H4, DfrB-H5 and DfrB-H6 provided TMP resistance up to the highest concentration of TMP soluble in 5% methanol (600 µg ml^−1^) (figure 3*a*; electronic supplementary material, table S2).

We then conducted assays for dihydrofolate reductase activity using cell lysate from the recombinant *E. coli* strains expressing the DfrB-H. Significant activity was detected only in the lysate of cells expressing DfrB-H2 and DfrB-H5 (figure 3*b*). Therefore, DfrB-H2 and DfrB-H5 showed both dihydrofolate reductase activity in bacterial lysate and TMP resistance *in vivo*. By contrast, the TMP resistance observed in DfrB-H4 and DfrB-H6 using the more sensitive MIC assay is consistent with dihydrofolate reductase activity too low to be detected in the cell lysates. Consistent with this hypothesis, the DfrB-H2 and DfrB-H5 proteins are readily observed upon overexpression, lysis and resolution by tricine-sodium dodecyl sulfate-polyacrylamide gel electrophoresis (SDS-PAGE), whereas the DfrB-H3, DfrB-H4 and DfrB-H6 proteins did not express at levels high enough to be visualized (electronic supplementary material, figure S10).

### (c) Extracting the homologous SH3-like segments from the DfrB-H

To better evaluate the similarities between the SH3-like fold of the DfrB-H proteins and DfrB1, we generated 78-residue segments named DfrB-H-Seg. The DfrB-H-Seg proteins are composed of the predicted SH3-like fold of the DfrB-H, preceded by the 20-residue N-terminus of DfrB1 (electronic supplementary material, figure S9). In the case of DfrB1, this N-terminus was shown to be essential for expression of the well-folded active protein, although its subsequent removal does not abrogate activity [36]. Indeed, all DfrB-H-Seg (except for DfrB-H3) could be observed on tricine-SDS-PAGE upon overexpression (electronic supplementary material, figure S10). Notably, the removal of the additional domains resulted in visible protein expression of DfrB-H4-Seg and DfrB-H6-Seg, whereas the full DfrB-H4 and DfrB-H6 proteins were not detectable.

MIC assays in *E. coli* overexpressing these DfrB-H-Seg proteins revealed TMP resistance phenotypes similar to their respective full-length protein (figure 2*a*; electronic supplementary material, table S2), indicating that the resistance phenotype is independent of the additional domains in the DfrB-H proteins. This was also the case for DfrB-H3-Seg protein expression which failed to provide TMP resistance, paralleling lack of TMP resistance from the full-length DfrB-H3. This suggests that the lack of activity of DfrB-H3 does not result from its additional domains preventing homotetramerization, but rather from a lack of soluble expression.

The dihydrofolate reductase activity of DfrB-H6-Seg in cell lysate was as high as that of DfrB1, whereas DfrB-H5-Seg displayed lower activity (figure 3*c*). The differences in activity between the full-length proteins and their extracted domains may result from differing expression levels; in particular, DfrB-H6 lacked visible expression and activity whereas DfrB-H6-Seg appeared as a clear band and showed high activity (electronic supplementary material, figure S10). These results demonstrate that the SH3-like fold of the DfrB-H proteins can suffice to procure TMP-resistant dihydrofolate reductase activity.

### (d) Kinetics and inhibition of the DfrB-H5 distant homologue

Having determined that the 41.6 kDa DfrB-H5 and that the independently expressed 10.9 kDa SH3-like fold DfrB-H5-Seg display similar activity in lysate, we compared their kinetic parameters (*k_cat_*, $K_{M}^{\mathrm{DHF}}$, $K_{M}^{\mathrm{NADPH}}$) to the 11.0 kDa DfrB1 reference protein. DfrB-H5 and DfrB-H5-Seg exhibit similar kinetic parameters as DfrB1 (table 1), demonstrating that the dihydrofolate reductase activity of DfrB-H5 is entirely defined by its SH3-like fold. The Y69L substitution has been shown to reduce ligand binding and decrease the rate of catalysis in DfrB1 [43]. To further investigate the mechanistic similarities between DfrB1 and DfrB-H5, their VQIY active-site motif was modified to VQIL. This produced a loss of TMP resistance and a significant reduction in dihydrofolate reductase activity in both DfrB1 and DfrB-H5 (table 1; electronic supplementary material, table S3). The analogous modulation of the DfrB1 and DfrB-H5 catalytic activities by this active-site substitution further supports mechanistic similarities.

**Table 1.** Kinetic parameters for the dihydrofolate reductase activity.

	$K_{M}^{\mathrm{DHF}}(\mu M)$	$K_{M}^{\mathrm{NADPH}}(\mu M)$	*k_cat_* (s^−1^)	$k_{cat}/K_{M}^{\mathrm{DHF}}(s^{-1}\mu M^{-1})$
DfrB1^a^	8.2 ± 0.11	1.6 ± 0.02	0.83 ± 0.01	0.10
DfrB-H2	8.7 ± 0.6	7.8 ± 0.6	1.35 ± 0.02	0.15
DfrB-H5	21 ± 7	12 ± 1	1.1 ± 0.2	0.05
DfrB-H5-Seg	30 ± 20	20 ± 4	3 ± 1	0.09
DfrB1-Y69L	400 ± 400	170 ± 20	0.03 ± 0.03	8.0 × 10^−5^
DfrB-H5 Y267L	NA^b^	NA	NA	NA

^a^Reference [44].

^b^Trace activity detected.

To further probe the structural similarities of the active sites of DfrB1 and DfrB-H5, we characterized the inhibition of dihydrofolate reductase activity of DfrB-H5 by representatives of two distinct classes of DfrB inhibitors. Inhibitor 1 belongs to a class of symmetrical bis-benzimidazoles that has been demonstrated to provide strong inhibition (*K*_i_ in the range of 2–62 µM) of DfrB enzymes by binding inside the active-site tunnel [38,49,50]. The second class of inhibitors (such as inhibitor 2) is composed of bisubstrate composite molecules formed from the DHF and NADPH substructures, and also effectively inhibit the DfrB enzymes (*K*_i_ 12–130 µM) [38]. Here, we show that DfrB-H5 and DfrB1 display essentially undistinguishable affinities for each inhibitor (table 2). Considering that the two classes of inhibitors are structurally unrelated, this finding is consistent with the binding region of DfrB-H5 being structurally analogous to that of DfrB1.

**Table 2.** Inhibition of DfrB-H5 with structurally distinct, DfrB-specific inhibitors. Inhibitor 1 is a bis-benzimidazole molecule, and inhibitor 2 is a bisubstrate molecule.

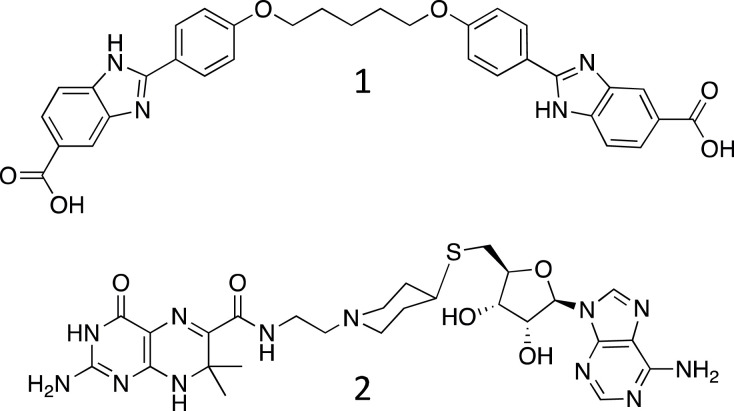				
	1		2	
	*K*_i_ (µM)	IC_50_ (µM)	*K*_i_ (µM)	IC_50_ (µM)
DfrB1	2.0 ± 0.3^a^	64 ± 11^a^	20 ± 3^b^	650 ± 87^b^
DfrB-H5	12 ± 6	60 ± 30	30 ± 10	170 ± 60

^a^Reference [49].

^b^Reference [38].

### (e) The DfrB-like domain promotes tetramerization

**Figure 4.**
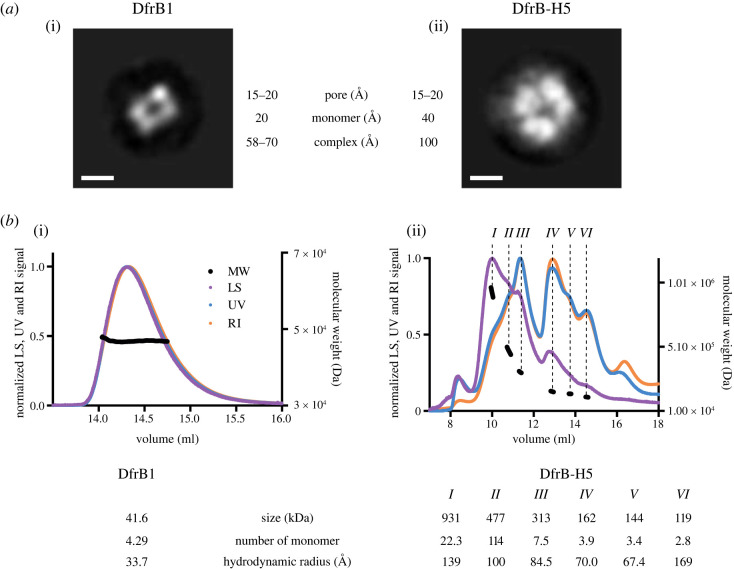
Oligomerization of DfrB-H5 analysed with two-dimensional classification and light scattering. (*a*) Negative-stain EM images for DfrB1 (i) and DfrB-H5 (ii) reveals homotetramerization of the proteins with pores of similar size. The images for DfrB1 and DfrB-H5 are a superposition of 1013 and 277 particles, respectively. The size of each monomer, overall complex and central pore is shown. The white bar corresponds to 50 Å. (*b*) Elution profile, with normalized light scattering data (LS) in purple, UV absorbance in blue and differential reflective index data (RI) in orange, of DfrB1 (i) and DfrB-H5 (ii) shown with the molecular weight (MW) estimated by MALLS in black. The size, the estimated number of monomer and the hydrodynamic radius are presented for each peak. (Online version in colour.)

The functional evidence above supports the hypothesis that the distant homologue DfrB-H5 forms an active site that is structurally analogous to that of DfrB1. We next investigated whether DfrB-H5 assembles into a homotetramer, which is known to be essential for the formation of functional DfrB1. We observe tetrameric arrangements of similar symmetry for both enzymes in negative-stain electron microscopy (EM), as well as a similar diameter for the single central tunnel (figure 4*a*). As expected, the DfrB-H5 tetramer (41.6 kDa per monomer) is 100 Å in diameter, which is significantly larger than the DfrB1 tetramer (58–70 Å; 11.0 kDa per monomer). These data support the formation of a symmetrical pore that is analogous to the functional DfrB-like active site.

DfrB1 assembled uniformly into apparent tetramers as evidenced by EM (figure 4). By contrast, two-dimensional class averages for DfrB-H5 were consistent with the formation of the tetrameric form (figure 4) as well as of dimers and trimers (electronic supplementary material, figure S11). We further investigated this multimeric assembly in solution, using analytical size exclusion chromatography-multi-angle laser light scattering (SEC-MALLS) [51] (figure 4*b*). Once again, DfrB1 formed highly homogeneous tetramers. By contrast, multiple heterogeneous peaks were detected in the case of DfrB-H5, with the main species being the tetrameric (3.9 monomers) and the octameric (7.5 monomers) forms. We also observed oligomeric species predicted to belong to dodecameric (11.4) and 24-mer (22.3) particles. Based on the higher order oligomers being multiples of 4 (8-, 12- and 24-mer), we hypothesize that these are complexes of tetramers. Other regions of the protein may contribute to formation of higher order oligomers that are observed. Taken together, results from EM and SEC-MALLS highlight the consistent assembly of DfrB1 into a functional homotetrameric form, whereas DfrB-H5 assembles into various oligomeric arrangements, consistent with the association of multiple tetramers.

In order to form its functional homotetrameric form, two unfolded DfrB1 monomers (M) dimerize; two dimers (D) then dimerize to form a tetramer (T), according to 4M⇌2D⇌T [36,48,52]. Each protomer contains a W38 at the dimer–dimer interface (figure 1) [48]. Each dimer–dimer interface also includes two H62 residues; as a result, DfrB1 is mainly in a dimeric state at pH 5 owing to protonation of H62 and is predominantly tetrameric at pH 8 [52,53]. The W38F substitution in DfrB1 impedes the formation of functional tetramer, resulting in 10- and 40-fold weaker effective binding to DHF and NADPH, respectively, and a 100-fold reduced catalytic turnover [48]. Nonetheless, the residual activity of W38F DfrB1 suggests a low level of tetramer formation.

Since W38 is conserved throughout the DfrB family and in all DfrB-H (electronic supplementary material, figure S8), the W38F substitution could serve to investigate similarities in the association mechanism of the SH3-like fold in these proteins. We began by further characterizing the phenotype and biophysical properties of W38F-substituted DfrB1. First, by means of native mass spectroscopy (MS), we confirmed that the W38F substitution significantly shifts the oligomeric populations of DfrB1, leaving a scarcely detectable population of tetramers (electronic supplementary material, figure S12). This trace of tetramers apparently provides the low amount of dihydrofolate reductase activity that is required to confer TMP resistance when overexpressed in *E. coli* (electronic supplementary material, table S3).

To determine whether assembly of the protomers into a tunnel-forming tetramer is analogous in DfrB1 and DfrB-H5, we mutated the conserved tryptophan of DfrB-H5 corresponding to W38 in DfrB1 (W236). We attempted to perform native MS measurements on DfrB-H5 and its resulting W236F variant; it was not possible to obtain signals, presumably owing to the poor ionization of the larger DfrB-H5 protein. Although the W236F substitution did not yield significant changes in the band pattern of DfrB-H5 on native PAGE (electronic supplementary material, figure S13), it gave rise to a TMP-sensitive phenotype (electronic supplementary material, table S3), indicative of a greater loss of dihydrofolate reductase activity than W38F DfrB1. This supports the hypothesis that the SH3-like fold of DfrB-H5 directs protein–protein interactions between protomers to form a tetramer analogous to that of DfrB1.

SEC analysis showed a clear shift of the main peak for DfrB-H5, centred at 1.31 ml, to 1.52 ml for W236F DfrB-H5, demonstrating a change in the predominant states of multimerization (electronic supplementary material, figure S14). In both cases, shoulders are observed on either side of the main peak. The poor separation of the species is consistent with conformational exchange within this population occurring on the timescale of the SEC experiment. Overall, this demonstrates that the conserved tryptophan plays a critical role in ensuring sufficient dihydrofolate reduction when DfrB-H5 is expressed in bacteria. The impact of the conserved W→F substitution is not identical in DfrB1 and DfrB-H5, which may indicate that other regions of DfrB-H5 participate in multimerization. Validation of this claim must await further studies.

Cumulatively, these biophysical data are compatible with a conserved role for the SH3-like fold in DfrB1 and in its distant homologue DfrB-H5. This region, characterized by high-sequence conservation (69% similarity between DfrB1 and DfrB-H5; electronic supplementary material, figure S7), appears to be sufficient to drive tetramerization even in the context of the much larger DfrB-H5 protein. The 58-residue SH3-like fold of DfrB-H5 is embedded within a larger protein architecture (residues 219 to 276 of a 365-residue protein), flanked by predicted *α* and *β* domains (figure 2*b*). The SEC-MALLS and the native PAGE data agree on the formation of defined, higher order oligomers. SEC-MALLS identifies the dominant forms of DfrB-H5 as being tetrameric and octameric, with dodecamer readily identified and a trace of 24-mer (figure 4*b*). This suggests the formation of tetramers, as well as dimers and trimers of tetramers (octamers and dodecamers), along with a trace of dimers of dodecamers. This is consistent with the SH3-like fold being the dominant force in multimer assembly, with other interactions of weaker strength potentially mediated by the other domains in DfrB-H5 not shared with DfrB1.

### (f) Biophysical properties of the SH3-like fold are similar in DfrB1 and DfrB-H5-Seg

**Figure 5.**
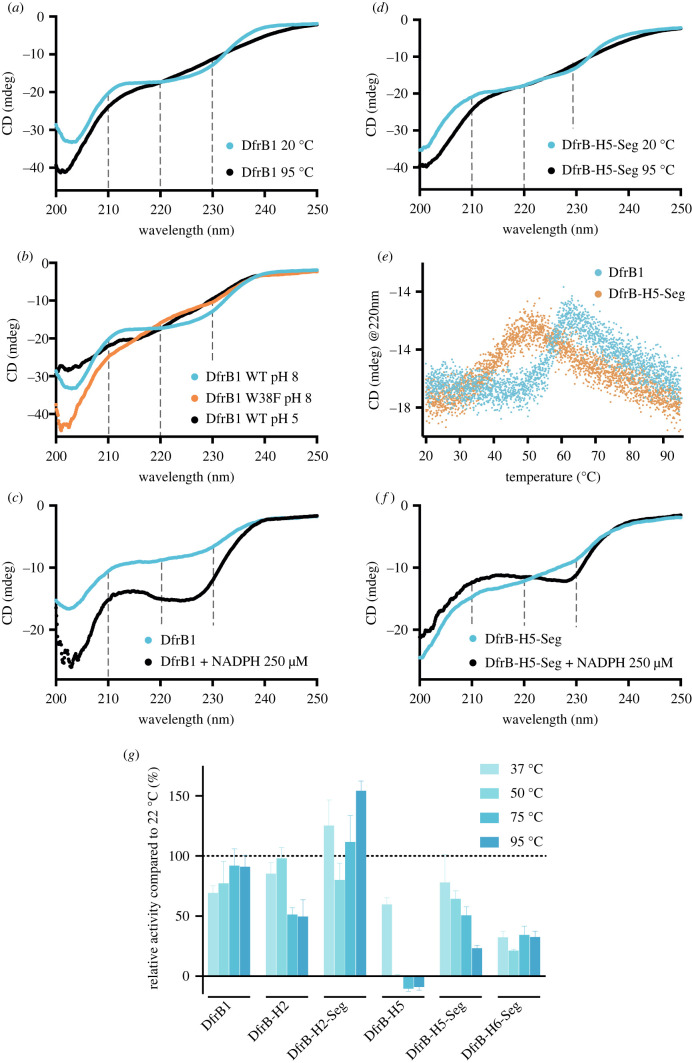
Influence of heating on multimerization and activity. (*a*) The 215–230 nm signature CD signal of DfrB1 at 20°C is lost when heated at 95°C. (*b*) The 215–230 nm signal is present only for DfrB1 at pH 8, when homotetramerization is possible. The W38F substitution in DfrB1 and pH 5 disrupt tetramerization. CD spectra at 20°C of DfrB1 (40 µM pH 8), DfrB1-W38F (40 µM pH 8) and DfrB1 (35 µM pH 5). (*c*) The 215–230 nm signal is stabilized when DfrB1 is incubated with NADPH. CD spectra of DfrB1 (25 µM) with and without incubation of NADPH 250 µM. (*d*) The CD spectra of DfrB-H5-Seg also display the 215–230 nm signature signal, comparable to DfrB1. The spectra at 95°C do not present the signature signal. (*e*) A signal change at 220 nm is observed at a lower temperature for DfrB-H5-Seg than DfrB1. A minor signal change occurs at 56.9 ± 0.2°C and 43 ± 1°C for DfrB1 and DfrB-H5-Seg, respectively. (*f*) CD spectra of DfrB-H5-Seg (25 µM) with and without incubation with 250 µM NADPH. The 215–230 nm signal is stabilized when DfrB-H5-Seg is incubated with NADPH. (*g*) Thermotolerance of dihydrofolate reductase activity for DfrB-H and DfrB-H-Seg proteins with detectable dihydrofolate reductase activity in cell lysate. (Online version in colour.)

Using circular dichroism (CD), we verified whether the extracted SH3-like segment of the distant homologues display similar properties. The His_6_-tagged DfrB1 includes 33% of structured regions and 67% of loops and the unstructured N-terminus. Consistent with this, DfrB1 shows two characteristic shoulders at 210 and 240 nm with a broad minimum between 215 and 225 nm at 20°C (figure 5*a*). The minimum at 203 nm is characteristic of the unstructured regions of DfrB1. Heating to 95°C induced little change other than a loss in definition (figure 5*a*). Considering the many factors that can cause a signal change upon heating an oligomeric enzyme, we hypothesized that this 215–230 nm signal is a signature of homotetramerization. Indeed, the CD spectrum at 20°C of DfrB1-W38F and DfrB1 at pH 5, both impeded in tetramer formation [48], did not exhibit this signal (figure 5*b*).

The SH3-like fold of DfrB-H5-Seg presented a similar spectral signature between 210 and 240 nm at 20°C and a similar loss of definition upon heating (figure 5*d*). This further supports analogy in the mechanism of tetramerization of the SH3-like fold in DfrB1 and DfrB-H5. Nonetheless, the *T*_m (220 nm)_ shifted from 56.9°C in DfrB1 to 43°C in DfrB-H5-Seg, demonstrating that its homotetramer is less thermostable than that of DfrB1 (figure 5*e*).

Ligand binding often stabilizes protein structure [54,55]. We investigated whether incubation of DfrB1 with NADPH and folic acid (the air-stable analogue of DHF), resulted in a modification of the CD signal, which could indicate a change in the equilibrium of the oligomeric species. In DfrB1, NADPH and DHF binding are mediated by residues from more than one protomer within the tetrameric tunnel, as observed by crystallography [41] (figure 1). Where the weak binding of folic acid (*K*_D_ 120 µM [56]) with DfrB1 did not modify the CD signal (data not shown), the strong binding of NADPH (*K*_D_ 2.5 µM [56]) sharpened the 210 nm shoulder and accentuated the local minimum at 225 nm (figure 5*c*). The DfrB-H5-Seg protein displayed a parallel, albeit weaker pattern upon NADPH binding (figure 5*f*). This is consistent with a similar NADPH-binding mechanism in DfrB1 and DfrB-H5.

DfrB1 is known to tolerate incubation at high temperature [38,57]. Other members of the DfrB family display similarly high recovery of activity following heating to 95°C and cooling (electronic supplementary material, figure S15). In fact, DfrB enzymes can be purified from lysate using incubation at 70°C [38]. We investigated whether the DfrB-H and their extracted segments producing detectable dihydrofolate reductase activity (figure 3*b*,*c*) exhibited similar thermotolerance. DfrB1 tolerated heating to 95°C for 10 min, recovering 91% activity upon cooling (figure 5*g*). DfrB-H2 displayed lower thermostability (50% activity recovered after heating to 75°C and 95°C) as a result to the 19 residues added to its 20-residue N-terminus. This is supported by the observation that the extracted segment of DfrB-H2, DfrB-H2-Seg, is thermostable, consistent with it differing from DfrB1 by a single residue. By contrast, DfrB-H5 lost activity in lysate following heating to 50°C or more. This can be attributed to the additional domains of the 365-residue DfrB-H5. Nonetheless, the extracted SH3-like fold of DfrB-H5, DfrB-H5-Seg, displayed considerable tolerance to heating, as did DfrB-H6-Seg. They respectively recovered 23% and 33% of their initial activity following heating to 95°C (figure 5*g*). The SH3-like fold of each of these proteins thus displays a pattern of heat tolerance reminiscent of that of DfrB1 yet with distinctive features that reflect their sequence differences (electronic supplementary material, figure S8).

## 3. Discussion

The TMP-resistant DfrB proteins were first detected on plasmids of pathogenic bacteria in the 1970s [58–60]. They have since been described as primitive enzymes, as various lines of evidence demonstrate that their catalytic mechanism had not been optimized by evolution [35]. A crucial indicator of this primitive mechanism is the absence of a catalytic acid. As a result, the catalytic mechanism relies on protonation of the DHF-N5 (p*K*_a_ = 2.59 [61]) by the solvent (electronic supplementary material, figure S4). This demonstrates a lack of evolutionary fine-tuning, in contrast with the catalytic mechanism of the ubiquitous FolA dihydrofolate reductase family, where a conserved aspartate or glutamate increases the p*K*_a_ of DHF-N5, facilitating the hydride transfer from NADPH to the imine [62].

Databases of characterized proteins yielded no protein homologous to the DfrB family using standard search tools. This begs the question: how has the DfrB family evolved to emerge in the modern resistome? We turned to the investigation of putative proteins to establish a potential evolutionary link with the DfrB family. Our search in the UniProtKB database, which includes putative proteins, yielded only 30 non-redundant proteins with sequence similarity to DfrB1, among which 12 were distant homologues. The predicted SH3-like fold of DfrB-H2 to DfrB-H6 share between 67 and 100% sequence similarity with DfrB1, suggesting common ancestry. Not only is high sequence similarity shared between the ß-strands of the SH3-like fold, but both the length and sequence are conserved in the inter-strand loops. This is notable, as inter-strand loops of SH3-like folds tend to differ greatly [32].

Remarkably, four of the five DfrB-H, sharing between 10 and 80% global sequence similarity with DfrB1, displayed the same high TMP resistance phenotype. The DfrB-H5 homologue (sharing 14% global sequence similarity and 63% local sequence similarity with DfrB1) displayed clear catalytic activity as well as numerous structural and functional similarities with DfrB1. These include similar pore size resulting from homotetramerization, the importance of the conserved VQIY motif and K32 to bind the negatively charged groups of DHF and NAPDH, and the similar inhibition of both enzymes by two structurally distinct molecules. The primitive catalytic mechanism is probably proximity based, with the active site orienting the reactive groups of DHF and NADPH for the hydride transfer event [41].

We have thus demonstrated that the DfrB-H proteins, consisting of a predicted SH3-like fold homologous to DfrB enzymes and a variety of additional structural domains, can provide the same antimicrobial resistance phenotype as the DfrB family. Their core architecture is compatible with dihydrofolate reduction by a DfrB-like mechanism. This suggests that the ancestors of the DfrB family could have displayed adventitious dihydrofolate reduction activity embedded in the context of varied and complex protein architectures of yet unknown function. The selective pressure recently provided by TMP could have promoted the extraction of the DfrB-like domain and its integration into the resistome by means of mobile genetic elements. This hypothesis is supported by the extraction of the SH3-like fold of the DfrB-H, where DfrB-H4-Seg, DfrB-H5-Seg and DfrB-H6-Seg display the same phenotype as the DfrB1. This demonstrates that the SH3-like fold of distant DfrB homologues can suffice to provide the phenotype required for survival when challenged with TMP. This is also consistent with the N-terminal extension of DfrB-H2 relative to the nearly identical though shorter DfrB1. In this case, it suggests that recent duplication and diversification of DfrB-H2 could have led to the second Met acting as the only start codon, yielding the 78-residue products that define the modern DfrB family.

Despite having uncovered structural and functional links between the DfrB and the diverse homologues, we have not accrued sufficient information to gain clear insight into the evolutionary origin of the DfrB family. The DfrB-H characterized here have no known native function nor evolutionary background, as searches for their homologues in UniProtKB/Swiss-Prot using PSI-BLAST yielded no significant hit aside from known DfrB members. Although none of the distant DfrB-H belongs to the same structural or evolutionary family, their high-sequence homology within the SH3-like fold supports a relation resulting from divergent evolutionary relationship (rather than convergent evolution) for the DfrB-like domain. We envision that tapping into the information captured in metagenomic databases will be essential to recapitulate the evolutionary path of the DfrB family towards the modern resistome.

## 4. Methods

### (a) Identification of the homologues

The homologous sequences were gathered from UniProtKB by searching for the Pfam family designation 06442 [UniProt release 2018_06] [63,64]. The resulting dataset of 68 sequences (30 non-redundant sequences) ranged from 67 to 463 amino acids in length. Non-redundant sequences were aligned with MAFFT [46]. The phylogenetic tree was generated using the alignment of the SH3-like sequence (positions 24 to 78 in DfrB1) using IQ-Tree (ultrafast bootstrap analysis, 1000 alignments) [65]. The tree was represented using iTOL [66]. Structure prediction was performed by ColabFold, using the relaxed option [45].

Five sequences, named DfrB-H, were selected from multiple sequence alignment and phylogenetic reconstructions, to ensure broad sampling of the Pfam06442 sequence space [67,68]. These sequences were codon optimized for *E. coli* expression and synthesized for cloning purposes.

### (b) Cloning

The DfrB-H, all with a N-terminal His_6_ tag, were ordered from TwistBioscience in pET28a vectors. Subcloning of cTEM-19 m was performed as previously described [69]. His-tagged DfrB3, DfrB5 and DfrB7 were subcloned in pET24 as previously reported [38], and His_6_-DfrB4 was obtained as reported previously [50]. DfrB1 with and without His_6_ in pET24 was obtained as previously reported [38,50]. Otherwise indicated, all mentions of DfrB1 refer to His_6_-DfrB1. Mutations W38F and Y267L were, respectively, introduced into DfrB1 and DfrB-H5 with Phusion Plus polymerase (Thermo) in a two-step reaction (1 min at 98°C, 30 cycles of 15 s at 98°C and either 2 or 3 min at 72°C, 5 min at 72°C). The primers used are as follows: DfrB1-W38F-F (5′-CGCCGCCTTCCAAGGTCAGATTG-3′), DfrB1-W38F-R (5′-CCGGATTTCTTGCGCACGCG-3′), DfrB-H5-Y267L-F (5′-TGTCCAAATTTTGCCGATCGCAGC-3′) and DfrB-H5-Y267L-R (5′-CTACCAGGTTCACGTTCTGACTCG-3′). Templates were digested using DpnI (NEB) O/N at 37°C. Mutations Y69L and W236F were, respectively, introduced in DfrB1 and DfrB-H5 using the QuickChange Lightning kit, with either 3 min or 3 min 40 s elongation time. The primer used was designed according to the kit: DfrB1-Y69L-F (5′-GCTCAGTACAGATTTTACCTGTTGCGGCGCTTGAACGCA-3′), DfrB1-Y69L-R (5′- GCGCCGCAACAGGTAAAATCTGTACTGAGCCTGGG-3′), DfrB-H5-W236F-F (5′-CGCAAAACTAAAGGTTCTAGTTTCCAGGGAGTAGTGG-3′) and DfrB-H5-W236F-R (5′-CTACTCCCTGGAAACTAGAACCTTTAGTTTTGCGCAC-3′). Reactions were transformed into chemically competent *E. coli* DH5*α*.

DfrB-H2-Seg, DfrB-H3-Seg and DfrB-H6-Seg were generated using a modified version of restriction-free cloning [70]. First, the pET24 backbone with the first 20 amino acids of the DfrB1 gene was amplified using 100 ng of template and following a three-step protocol (1 min at 98°C, 18 cycles of 30 s at 98°C, 50 s at 5°C below melting temperature and 1 min kb^−1^ at 68°C and 10 min at 68°C) using the PfuUltra polymerase (Agilent). The following primers were used: pET24-F (5′-TAAAAGCTTGCGGCCGCACTC-3′) and Nterm-R (5′-CGATGGGAATACAAAATTGCCAGCAAC-3′). Then, the megaprimer was generated by amplifying the segments of DfrB-H3 and DfrB-H6 with a three-step protocol (30 s at 98°C, 30 cycles of 30 s at 98°C, 30 s at 55°C and 20 s at 72°C, and 10 min at 72°C) using the Phusion polymerase (Thermo). The primers used are as follows: DfrB-H2-Seg-F (5′-CCAAGACTACAAAGACGATGACGACAAGATGGAACGTTCTAGCAATGAGG-3′), DfrB-H2-Seg-R (5′-GTGCGGCCGCAAGCTTTTAATTTATGCGTTCCAAGGCTGC-3′), DfrB-H3-Seg-F (5′-GTTGCTGGCAATTTTGTATTCCCATCGCAGGGAAAATTCCGCATGG-3′), DfrB-H3-Seg-R (5′-CGAGTGCGGCCGCAAGCTTTTACATCCACTGACGCCATCT-3′), DfrB-H6-Seg-F (5′-GTTGCTGGCAATTTTGTATTCCCATCGGTGGGCAAATTTCAGCGAG-3′) and DfrB-H6-Seg-R (5′-CGAGTGCGGCCGCAAGCTTTTAATGTGAAAGCCGAAGGGC-3′). Templates were digested by DpnI (NEB) for 2 h at 37°C and reactions were cleaned using the Monarch PCR & DNA Cleanup Kit (NEB). With the cleaned megaprimers and the pET24 backbone, the DfrB-H-Seg were assembled using the two-step secondary PCR protocol from rf-cloning.org [71]. Reactions were cleaned using the Monarch PCR & DNA Cleanup Kit (NEB) and transformed into chemically competent *E. coli* DH5*α*. DfrB-H4-Seg and DfrB-H5-Seg were ordered from TwistBioscience in pET24. All sequences were confirmed by DNA Sanger sequencing (Genome Quebec platform at Sainte-Justine Hospital).

### (c) Minimal inhibitory concentration

MICs were determined in triplicates according to Wiegand *et al*. [72] using both the agar and broth microdilution method. Briefly, *E. coli* BL21(DE3) cells expressing DfrB-H, DfrB-H-Seg, positive control DfrB1 and negative control cTEM-19 m [69] were propagated overnight in Luria-Bertani (LB) media with 50 µg ml^−1^ kanamycin. For the agar method, an inoculum of 10^4^ colony-forming units (cfu) was spotted on LB agar plates with 0.25 mM IPTG (ThermoFisher) and TMP (Sigma) in twofold concentration steps up to 600 µg ml^−1^; the latter is the highest concentration of TMP soluble in a final concentration of 5% methanol. The TMP concentration inhibiting bacterial growth following overnight incubation at 37°C was considered to be the MIC. For the broth method, in 96-well plates, LB media was inoculated with 10^5^ cfu ml^−1^, with 0.1 mM IPTG and TMP. MICs were determined as described above.

### (d) Dihydrofolate reductase activity in lysate

The various DfrB proteins (DfrB1, DfrB-H and DfrB-H-Seg) and negative control cTEM-19 m were overexpressed in *E. coli* BL21(DE3) as follows. An overnight LB 50 µg ml^−1^ kanamycin preculture was used to inoculate 10 ml LB cultures to an optical density (OD)_600nm_ of 0.1 for DfrB-H, DfrB-H-Seg and their controls. Five microliters cultures of DfrB-H and their controls were incubated at 37°C for 3 h, followed by overnight 1 mM IPTG induction at 30°C, 230 rpm. Cultures of DfrB-H-Seg and their controls were incubated at 37°C for 3 h, followed by 3 h 1 mM IPTG induction at 37°C, 230 rpm. As for the other DfrB proteins, 10 ml culture was prepared in auto-induction media ZYP-5052 (928 ml ZY (1% tryptone, 0.5% yeast extract), 50 ml 20×P (50 mM Na_2_HPO_4_, 50 mM KH_2_PO_4_, 25 mM (NH_4_)_2_SO_4_), 20 ml 50 × 5052 (0.5% glycerol, 0.05% glucose, 0.2% α-lactose), 2 ml MgSO_4_ (2 mM) and 0.2 ml 1000 x trace elements (0.2x)) and incubated at 37°C up to an OD_600nm_ of 0.1, followed by an overnight induction at 22°C, 230 rpm. The cultures were centrifuged at 12 800*g* for 30 min at 21°C and the pellets were stored at −72°C. Cell pellets were thawed at room temperature (RT) for 30 min and resuspended in 600 µl of lysis buffer (0.1 M potassium phosphate buffer pH 8, 10 mM MgSO_4_ (Anachemia), 1 mM dithiothreitol (Fisher), 0.5 mg ml^−1^ lysozyme (MP Biomedicals), 0.4 U DNAse (Thermo), 1.5 mM benzamidine (Fisher) and 0.25 mM phenylmethylsulfonyl fluoride (Bioshop). Cells were incubated at RT for 2 h with vigorous shaking. Following centrifugation at 20 800*g* for 30 min at 21°C, 100 µl of the respective supernatants were transferred to 0.2 ml flat cap PCR (Fisher) tubes and incubated at different temperatures for 10 min, followed by 10 min on ice. The heated lysates were centrifuged at 12 800*g* for 15 min at 21°C. The supernatants were resolved in 10% tricine-SDS-PAGE gels to visualize the protein content of each variant incubated at various temperatures.

DHF (synthesized as previously reported [73]) and NADPH (Chem Impex) were quantified spectrophotometrically in 50 mM pH 7 potassium phosphate buffer ($\varepsilon_{282\mathrm{nm}}^{\mathrm{DHF}}$ 28 400 M^−1^cm^−1^ and $\varepsilon_{340\mathrm{nm}}^{\mathrm{NADPH}}$ 6200 M^−1^cm^−1^). In a 96-well UV transparent plate (Corning), 10 µl of lysate was added to 100 µM NADPH and 100 µM DHF in 50 mM potassium phosphate buffer pH 7 for a final volume of 100 µl. Enzyme activity was determined by monitoring the depletion of DHF and NADPH at 340 nm with a plate reader (Beckman Coulter DTX 880). Initial rate of the reaction was determined on the first 20% of reaction (substrate conversion to product) with the depletion of NADPH and DHF at 340 nm (Δ*ε*_340nm_ 12 300 M^−1^cm^−1^ to determine product formation). Assays were carried out in triplicate**.**

### (e) Protein expression and purification

Expression of DfrB1, DfrB-H and DfrB-H5-Seg transformed in *E. coli* BL21(DE3) was carried out as follows. Overnight precultures of 5 ml inoculated 500 ml of Terrific Broth media containing 50 µg ml^−1^ kanamycine (Sigma). After initial growth at 37°C up to OD_600nm_ of 0.6, cells were induced by 1 mM IPTG (Thermo) and expression was carried out either at 30°C or 37°C overnight, with the exception of DfrB-H5-Seg (expression for 3 h only). The cells were harvested and resuspended in IMAC A buffer (600 mM NaCl, 50 mM Tris, 1 mM CaCl_2_, 20 mM imidazole and pH 8), lysed with a cell disrupter (Constant Systems) and centrifuged at 16 000*g* (Sorvall SLA-3000) at 4°C for 30 min. The supernatant was then either filtered with a 0.2 µm filter, injected onto a HisTrap FF column (Cytiva) and eluted with IMAC B buffer (600 mM NaCl, 50 mM Tris, 1 mM CaCl_2_, 500 mM imidazole and pH 8), or directly incubated with Ni-Profinity IMAC Resine (BioRad), eluted using IMAC B buffer and further purified using a Superdex 75 column (1.6 × 55 cm) equilibrated with 50 mM pH 8 potassium phosphate buffer. Buffer exchange and concentration of protein fractions were carried out with Amicon Ultra Centrifugal Filter Units of 3 K or 30 K molecular weight cut-offs (Fisher).

Expression and purification of DfrB1 without a histidine tag in *E. coli* BL21(DE3) was performed as follows. An overnight preculture was used to inoculate 600 ml (3 × 200 ml) of auto-induction ZYP-5052 media containing 50 µg ml^−1^ kanamycin. The culture was incubated at 37°C for 3 h, followed by an overnight induction at 22°C. The cells were harvested and resuspended in 30 ml of pH 8 potassium phosphate buffer, lysed with a cell disruptor and centrifuged at 16 000*g* at 4°C for 25 min. The supernatant was heated at 75°C for 10 min and then cooled on ice for 10 min. The heated lysate was centrifuged at 12 800*g* at 4°C for 20 min. The supernatant was concentrated with an Amicon Filter, filtered and injected into the Superdex 75 column. Pure fractions were pooled together and concentrated.

The mass of each purified protein was confirmed by the Regional Mass Spectrometry Centre at Université de Montréal.

### (f) Kinetic parameters *K*_M_ and *k*_cat_

DHF and NADPH were quantified as described in the section 'Dihydrofolate reductase activity in lysate'. Kinetic assays were performed in 1 cm pathlength quartz cuvette at 27°C in a Cary 100 Bio UV-Visible (Agilent) spectrophotometer by monitoring the initial rate of linear depletion of NADPH and DHF at 340 nm (*Δε*_340nm_ 12 300 M^−1^ cm^−1^ [74]) in 50 mM pH 7 potassium phosphate buffer. For the determination of $K_{M}^{\mathrm{DHF}}$ and $K_{M}^{\mathrm{NADPH}}$, the concentration range of the variable substrate span from 3.125 to 145 µM. The second substrate was kept at a saturating concentration of 50 µM, except for DfrB1-W38F and DfrB-H5-W236F for which it was kept at 100 µM. Data were fitted to the Michaelis-Menten equation using nonlinear regression analysis, with the exception of DfrB1-W38F which was fitted to the Lineweaver-Burk representation, using GraphPad Prism version 7 for Mac (GraphPad Software, San Diego, CA, USA). Standard deviation is shown.

### (g) Inhibition assays

Inhibitor **1** (2,2′-[1,5-pentanediylbis(4-oxyphenylene)]-bis-1H-benzimidazole-5-carboxylic acid) [49] and **2** (2-amino-N-(2-(4-((((2S,3S,4R,5R)-5-(6-amino-9H-purin-9-yl)-3,4-dihydroxytetrahydrofuran-2-yl)methyl)thio)piperidin-1-yl)ethyl)-7,7-dimethyl-4-oxo-3,4,7,8-tetrahydropteridine-6-carboxamide) [75] were dissolved in dimethyl sulfoxide (DMSO) and 50 mM pH 7 potassium phosphate buffer, respectively, to prepare stocks of 10 mM. Both inhibitors were subsequently diluted to the appropriate concentrations for the inhibition assay (0–400 µM). The inhibition assay of DfrB-H5 consisted of 50 µM DHF and 50 µM NADPH in a final volume of 100 µl of 50 mM pH 7 potassium phosphate buffer, in addition of the diluted inhibitor. The inhibition assay of **1** was performed in 10% DMSO. The reaction was initiated by adding approximately 0.006 mg of purified DfrB-H5 to the reaction mix. The detection of enzyme activity was described above. The IC_50_ values were determined with GraphPad Prism using the log[inhibitor] versus response (four parameters) equation, using a 95% confidence interval to establish error. The *K*_i_ constant for the respective substrates was calculated using the Cheng-Prusoff equation [76]:

$$K_{i}=\;\frac{IC_{50}}{1+([NADPH]/K_{M}^{NADPH})}.$$

### (h) Negative-stain electron microscopy sample preparation

Purified protein from a single and homogeneous gel filtration peak was diluted to a concentration of 40 ng µl^−1^. The sample (3 µl) was applied on a glow-discharged 300 mesh copper grid for 1 min and negatively stained with three consecutive droplets of 0.75% (w/v) uranyl formate solution (Electron Microscopy Sciences). The grid was blotted with Whatman filter paper to remove staining excess and air-dried at RT.

### (i) Electron microscopy data collection and analysis

Data were acquired using FEI Tecnai T12 120 kV transmission electron microscope equipped with a FEI Eagle 4k×4k CCD camera at a magnification of 67 000 × with a pixel size of 1.65 Å for DfrB-H5 and a magnification of 110 000 × with a pixel size of 0.98 Å for DfrB1. Each image was acquired using a 1 s exposure time with a total dose of 50 e^−^Å^−2^ and a defocus of −1.3 µm. Two-dimensional classification was performed using cryoSPARC [77]. CTFFIND4 (Wrapper) was used for the contrast transfer function estimation [78].

### (j) Size exclusion chromatography-multi-angle laser light scattering

Absolute molar mass was calculated using the ÄKTAmicro system (GE Healthcare) coupled with a Dawn HELEOS II MALLS detector and an OptiLab T-rEX online refractive index detector (Wyatt Technology). Five hundred microliters of protein sample was injected onto the Superdex 200 10/300 GL HPLC size exclusion column (Cytiva) for DfrB-H5 and Superdex 75 10/300 GL (Cytiva) for DfrB1 at a flow rate of 0.4 ml min^−1^. Bovine serum albumin was used for calibration.

### (k) Native mass spectrometry

DfrB1 WT and its W38F mutant were buffer exchanged into 200 mM ammonium acetate (MS grade) pH 7.5 using Micro Bio-Spin 6 columns (BioRad). Platinum-coated borosilicate nanospray emitters were prepared in-house as described previously [79] and were used to electrospray 10 µM of buffer-exchanged proteins on a Synapt G2-Si ion mobility mass spectrometer (Waters) equipped with a nanospray electrospray ionization source. MS data for both proteins were acquired in positive ion and sensitivity modes using the following instrumental settings: capillary voltage = 1.5 kV, cone voltage = 50 V, source offset = 50 V, source temperature = 65°C, trap gas (argon) flow = 2 ml min^−1^ and trap DC bias = 35 V. For ion mobility measurements, the following settings were used: ion mobility spectrometry (IMS) gas (nitrogen) flow = 90 ml min^−1^, IMS bias = 3 V, IMS wave velocity = 550 ms^-1^ and wave height = 40 V. Data were collected in triplicate for both proteins.

### (l) Native gel

The quaternary structure of purified proteins was analysed with clear native polyacrylamide gel electrophoresis (CN-PAGE) using pH 7, 4% to 16% Bis-Tris NativePage gels (Thermo) as previously described [80]. Briefly, protein samples were prepared in a loading dye composed of 50 mM Bis-Tris, 5% bromophenol blue (Fisher) 500 mM 6-aminocaproic acid and 10% glycerol. Following loading, native electrophoresis was performed at 4°C using a cathode buffer composed of 50 mM tricine and 15 mM Bis-Tris, pH 7, and an anode buffer of 50 mM Bis-Tris, pH 7. Following migration, gels were stained with Coomassie brilliant blue R-250 and destained with 10% acetic acid and 45% methanol.

### (m) Size exclusion chromatography

The oligomerization states of DfrB-H5 WT and W236F were analysed using analytical SEC with an ÄKTA fast protein liquid chromatography system. The 2.4 ml size exclusion column (Superdex 200 Increase 3.2/300, Cytiva) was calibrated with the Cytiva Gel Filtration Calibration Kit. Different protein concentrations (10 µl injections) were applied onto the column equilibrated with 50 mM potassium phosphate, pH 8, at a flow rate of 0.075 ml min^−1^.

### (n) Circular dichroism

Far-UV (250–200 nm) CD protein spectra were recorded at 20°C and 95°C using a JASCO J-815 spectropolarimeter, in a 1 mm optical path length cuvette. Denaturation spectra were performed at 220 nm, heating from 20 to 95°C with an increase of 1°C min^−1^. Protein samples (40 µM unless otherwise indicated) were prepared in 200 µl of 50 mM potassium phosphate buffer (pH 8, unless otherwise indicated). The temperature was regulated using a Peltier-type JASCO CDF-426S/15 thermostatic controller. All experiments were performed in triplicate. The data were corrected from the background and extracted using the Spectra Manager Suite (JASCO). Mean values and s.e. of the CD spectra were analysed and plotted using GraphPad Prism 7.0. The denaturation graphs present all data points from the triplicate measurements.

### (o) Thermotolerance assay

The initial rate of the reaction for each protein was independently determined for the various incubation temperatures. The relative activity (RA) for each enzyme was determined by comparing the initial rate of each temperature to the initial rate of the reaction at RT (RA (%) = initial rate incubated T°C / initial rate at 22°C *100). Data were analysed with GraphPad Prism 9.

## Data Availability

The data are provided in the electronic supplementary material [81].
